# Supplementary material for: Are carbon emissions trading and green financial instruments synergistic? -Comprehensive quantitative research based on content analysis
Source: PLoS One. 2024 Mar 7;19(3):e0298601. doi: 10.1371/journal.pone.0298601 (PMC10919601; doi:10.1371/journal.pone.0298601)
Supplement: S1 Data — (ZIP) [file pone.0298601.s001.zip › Data/地级市Z_F工作报告.zip!/║┌┴·╜¡/╞δ╞δ╣■╢√╩╨/└┤╫╘╣1⁄2╓┌║┼í╛┬φ┐╦╩2╛▌═°í┐_2017.docx]

各位代表：

　　现在，我代表市十五届人民政府，向大会报告过去五年的工作，对今后五年及2017年工作目标任务提出建议，请予审议，并请各位政协委员提出意见。

　　一、过去五年工作回顾

　　五年来，我们认真贯彻党中央、国务院和省委、省政府的战略部署，按照省实施“五大规划”、发展“十大重点产业”、建设“龙江丝路带”的总体要求，在市委的领导下，践行“五大发展理念”，主动适应把握新常态，克服经济下行压力，确定并实施12个重点产业，着力稳增长、调结构、促改革、惠民生，实现了“十二五”胜利收官和“十三五”良好开局。2016年，预计全市实现地区生产总值1350亿元，年均增长6.6%；社会消费品零售总额750亿元，年均增长12.4%；固定资产投资920亿元，年均增长12.3%；城乡居民人均可支配收入达到24630元和12890元，年均增长9.5%和10.9%；万元GDP综合能耗累计下降34.8%；主要污染物减排完成省下达计划。公共财政预算收入完成76.3亿元，按可比口径年均增长6.5%。

　　——农业综合生产能力明显提高。抢抓“两大平原”现代农业综合配套改革契机，加快推动传统农业向现代农业迈进。种植业生产体系逐步完善。积极推进土地规模经营，粮食产能稳定在250亿斤以上。绿色有机食品基地面积达到1400万亩，认证标志突破400个。成为国家级生态原产地产品保护示范区。落实“互联网+农业”省级示范基地370个。省级以上农民合作示范社和规范社达到158个。综合机械化程度达到95.9%。新增节水灌溉293.6万亩，治理水土流失178.6万亩，开发水田249.2万亩。畜牧业规模化发展逐步形成。畜牧业实现产值243.7亿元，比2011年增长55%。“两牛一猪”标准化规模养殖场达到1731个，规上畜牧业龙头企业达到32户，飞鹤、元盛、恒阳、丰源等畜产品加工企业不断壮大。农业产业化步伐逐步加快。农产品龙头企业发展到230户，加工转化率达43%，比2011年提高6个百分点。马铃薯产业发展迅速，中荷、龙科雪川等种薯繁育及马铃薯加工项目扎实推进。现代中药产业开始破题，4个县区建成万亩中药种植基地，安贞饮片加工等项目进展顺利。

　　——工业经济结构不断优化。加快调整产业结构、产品结构和所有制结构，全市规上工业企业达到367户，增加值年均增长5.9%。产业结构不断优化。轻重工业比调整到1.48:1。食品加工业迅猛发展，增加值年均增长12.4%，占规上工业比重达到56%。产品结构不断优化。规上工业企业研发新产品247项，其中123项列入省重点新产品研发计划，21项获得省重点领域首台套产品称号。新增国家级高新技术企业13户，新建省级以上工程技术研发中心22家、科技孵化器14个，科技成果转化1072项。所有制结构不断优化。地方规上工业占比提高到85%，非公经济占比提高到77%。推进央企与地方企业融合发展，一批地方企业成为一重等骨干企业战略合作伙伴。积极支持民营企业发展，助企周转贷款8.4亿元，发放助保金贷款12亿元。工业园区达到20个，入驻中小企业834户。28户企业享受直供电政策。

　　——服务业全面提升。第三产业增加值年均增长7.8%，生产性服务业比重提高7个百分点。商贸服务业持续发展。万达、兴隆大家庭等商贸综合体和星光科技等电商产业园建成运营。规上交易市场、限上企业、电商企业分别发展到37个、191户和139户。快递和电信业务量年均分别增长42.3%和23%。金融服务能力增强。金融业增加值年均增长18.5%；存贷款余额年均分别增长12.8%和21.9%。开展银税、银保合作；引进金融机构13家；5支产业引导基金相继设立；东北亚大宗商品、北方工业股权、北方畜产品3个交易中心建成运营；37户企业在新三板和区域市场挂牌。旅游、养老业势头强劲。旅游收入年均增长18.8%。水师森林温泉度假、碾子山国际滑雪场、奥悦水世界等一批新型旅游综合体建成运营，扎龙观鹤成为全省黄金旅游品牌。新晋3A级以上景区12家，新开通国内国际航线10条。新世纪老年公寓、中医百草养生院等50多家养老院投入运营，全市养老床位达到3.2万张。

　　——重点领域改革稳步推进。依靠改革，不断增强发展内生动力。经济领域改革持续深化。农业经营主体创新、农村金融、职业教育和地方国有粮食购销企业改革取得实效，农村行政村土地实测基本完成。黑化与昊华重组，厂办大集体改革和央企“三供一业”移交进展顺利。行政体制改革成效明显。建立四级政务服务体系，全面推行“三集中、三到位”审批，集中审批率达92%，市县两级审批事项分别精简67.5%和30%；“五证合一”商事制度改革全面完成。公共资源实行集中统一交易。“双随机、一公开”综合执法体制改革全面启动。其他改革取得积极进展。政府机构、事业单位分类、财税体制、政府投融资体制、医药卫生体制、不动产登记制度等改革均有新突破。

　　——城乡面貌发生深刻变化。统筹城乡规划、建设和管理，成功晋升国家级生态文明先行示范区和省级生态市。城乡居住条件明显改善。改造棚户区9.8万户，建成公（廉）租房8762套，城镇人均居住面积由25.6平方米增加到30.4平方米；改造老旧散小区437万平方米；既有建筑节能改造1033万平方米。改造农村危房19.8万户。拆除城区违章建筑1.4万处。基础设施建设逐步完善。新建改建“三供”管网1145公里。10座污水处理厂建成运行，新建垃圾处理厂12座。哈齐高铁建成通车，实施齐富嫩江公路大桥、滨洲公铁立交桥、中心城区南北互通等重点交通项目23项。升级改造市政主次干道360条，建设农村公路3138公里。南苑新城建设初具规模。劳动湖南扩基本完工。生态环境保护持续加强。节能减排示范市建设稳步推进。嫩江干流断面水质达标率达到87.5%。淘汰燃煤小锅炉1184台。新增清洁能源公交车483台。新增省级以上自然保护区5个，森林覆盖率和建成区绿化覆盖率分别提高到10.3%和40%。美丽乡村示范村和达标村达到770个。

　　——民生持续改善。坚持办好民生实事，切实维护群众利益。脱贫攻坚取得阶段性成果。投入专项扶贫资金11亿元，303个贫困村、37.8万贫困人口实现脱贫，其中2016年实现脱贫104个村、15.6万人，分别占贫困村和贫困人口总数的33.5%和60.9%。就业创业扎实推进。投入专项资金20.4亿元，支持就业创业。职业技能培训42.9万人次，发放创业贷款17.9亿元，实施援企稳岗，登记失业率控制在4.3%以内。建立创业孵化基地170个、众创空间20家。新增私营企业1.4万户、个体工商户5.7万户。社保水平大幅提升。城镇职工基本养老金人均提高75%。医保实现城乡统筹，城镇职工和城乡居民政策内平均报销比例达到90%和75%。城乡低保救助标准分别提高1.1倍和1.7倍；五保户集中和分散供养标准分别提高1.8倍和1.6倍；医疗救助比例提高1.6倍。民康医院等10家公益性福利机构建成使用。社会事业长足发展。5个县区通过义务教育基本均衡县验收；职业教育、艺体教育成绩斐然。城乡基层医疗卫生服务体系进一步完善，医疗床位达到2.8万张。全面两孩政策稳步实施。群众体育蓬勃发展，开展国际冰球等体育赛事300余项次，荣膺亚洲最佳冰球城市。文化惠民扎实开展，工人文化宫修缮使用。晋升为国家历史文化名城。社会保持和谐稳定。一批信访积案得到有效解决，社会治安、安全生产形势稳中向好。全国双拥模范城实现“七连冠”。

　　——政府自身建设得到加强。认真执行中央八项规定，深入开展群众路线、“三严三实”和“两学一做”系列教育，坚持不懈反“四风”，严格落实党风廉政建设“一岗双责”，强化监察和审计监督问责，政风转变成效明显。完善了政府议事决策规则，全面推行政府法律顾问制度，政府权力运行更加规范。清理腾退办公用房7.5万平方米，清理“吃空饷”人员920人，机关事业单位人员编制、“三公”经费得到有效控制。自觉接受人大、政协监督，议案和提案办复率100%。依法审修地方性法规草案7部、出台政府规章27部。“六五”普法圆满收官。

　　机关事务、新闻出版、社会科学、妇女儿童、民族宗教、食药监、地方志、物价、质监、统计、档案、人防、残联、气象、地震等各项工作，也都取得了新成绩。

　　各位代表！过去五年的成绩来之不易，这得益于省委、省政府和市委的正确领导，得益于市人大、市政协及各民主党派的监督支持，得益于全市上下的共同努力。在此，我代表市人民政府，向全市人民，向各级人大代表、政协委员，向各民主党派、工商联、无党派人士和人民团体，向驻齐解放军、武警官兵和中省直单位，向所有关心、支持齐齐哈尔改革发展的各界人士，表示衷心的感谢！

　　同时，我们也清醒地看到，政府一些工作完成得还不够好，全市经济社会发展还存在很多问题。在经济发展方面：市场化程度低，民营经济偏弱，结构不优，创新不够，人才匮乏，区域、行业间发展不平衡。一产供给质量不高，二产有效供给不足，三产供给力不强，严重影响实体经济振兴发展。在民生方面：居民收入总体偏低，就业再就业压力依然较大；看病难、看病贵问题尚未有效解决；教育质量和师德师风还需强化；城市公交发展滞后；一些小区物业管理服务水平有待提高；供水、供热还有薄弱环节；生态环境保护和治理任务艰巨。在政府自身建设方面：一些政府部门服务理念、法治观念、运转效能、诚信体系建设还需强化；个别公务人员对群众和企业“生冷硬”，甚至“中梗阻”“勒卡要”，发展环境还需改善；一些干部存在“只要不出事、宁可不干事”的思想倾向，不敢担当、不愿作为。对此，我们一定高度重视，认真加以解决。

　　二、今后五年及2017年工作目标任务

　　今后五年，是全面建成小康社会的决胜期，是全面深化改革的攻坚期，也是深入贯彻落实习近平总书记系列重要讲话特别是对黑龙江的两次重要讲话精神、推进齐齐哈尔全面振兴发展的机遇期。在新常态、新方位大背景下，稳中求进是我们当前和今后一个时期必须遵循的思路和方法。作为老工业基地，我市正处在转型发展的适应期和调整期，压力巨大，不进则退。但从总体上判断，机遇大于挑战，特别是党中央、国务院对东北地区的高度重视和亲切关怀就是最大的机遇。随着新一轮东北老工业基地振兴、“中国制造2025”、深化供给侧结构性改革、“一带一路”以及“龙江丝路带”战略的实施，支撑我市振兴发展的政策效应和有利要素正在逐步显现，全市上下聚焦经济建设的信心更加坚定。只要我们牢牢抓住这些宝贵的发展机遇，紧紧依靠市委的领导，保持定力，奋力前行，就一定能在齐齐哈尔全面振兴发展的伟大实践中，再造新优势，再创新辉煌！

　　按照市第十三次党代会总体部署，今后五年政府工作的总体思路是：高举中国特色社会主义伟大旗帜，深入学习贯彻习近平总书记系列重要讲话精神，遵照以习近平同志为核心的党中央战略部署，统筹推进“五位一体”总体布局和协调推进“四个全面”战略布局，落实省委创新实施“五大规划”发展战略，牢固树立新发展理念，适应把握引领经济发展新常态，以稳中求进为总基调，以提高发展质量和效益为中心，以推进供给侧结构性改革为主线，以做好“三篇大文章”、发展12个重点产业为支撑，着力构建新型产业体系，着力保障和改善民生，着力培育发展新动能，着力加强政府自身建设，奋力走出齐齐哈尔全面振兴发展的新路子，与全省同步全面建成小康社会。

　　今后五年的奋斗目标是：

　　经济发展质量效益稳步提高。新型产业体系初步形成。地区生产总值年均增长6.5%以上，规上工业增加值年均增长7%，固定资产投资年均增长8%左右，公共财政预算收入年均增长5%，社会消费品零售总额年均增长12%。

　　人民生活持续改善。脱贫攻坚取得全面胜利，城镇登记失业率控制在4.5%以内，城乡居民人均可支配收入年均分别增长8%和9%以上，群众获得感普遍增强。

　　生态建设成效显著。资源利用更加节约，生态保护和治理得到加强。万元GDP综合能耗累计下降13%，主要污染物排放量显著下降。

发展新动能全面发力。重点领域改革持续深化，对外开放不断扩大，“大众创业、万众创新”氛围更加浓厚。科技成果转化率达到80%。

　　2017年，按照中央、省委经济工作会议和市第十三次党代会要求，全力做好稳增长、促改革、调结构、惠民生、防风险各项工作，促进经济平稳健康发展和社会和谐稳定。主要预期目标是，地区生产总值增长6%以上，规上工业增加值增长5%左右，固定资产投资增长9%以上，公共财政预算收入增长2%以上，社会消费品零售总额增长10%，城乡居民人均可支配收入分别增长7%和8%，城镇登记失业率控制在4.5%以内，万元GDP综合能耗下降3.6%，主要污染物减排任务全面完成。

　　（一）深化供给侧结构性改革，加快构建新型产业体系。今后五年，把落实“三篇大文章”聚焦到12个重点产业上，深化供给侧结构性改革，打通一产供需通道，增强二产核心竞争力，发展三产新业态、新模式，推进三次产业融合，扩大高质量产品和服务有效供给，着力振兴实体经济，加快构建新型产业体系。2017年，重点做好以下工作：

　　推进质量效益型产业项目建设。以扩大有效供给为目的，大上质量效益型产业项目，全年推进固定资产投资5000万元以上产业项目超过200个。围绕国内国际有总需求增长空间和我市有供给竞争优势的领域谋划项目，突出以12个重点产业为核心的新型产业体系上项目，以项目开工率、竣工率特别是投产达效率为标准检验项目建设。按照“准备之冬、开工之春、建设之夏、竣工之秋”的推进方式，分阶段、分层次、分类别加快项目建设。以“中国制造2025”实施为契机，大力实施智能化改造类产业项目，推进装备制造整机、基础零部件、金属新材料等“老字号”转型升级；以“粮头食尾”“农头工尾”为抓手，深度开发资源深加工类产业项目，推进食品加工、马铃薯、现代畜牧以及日用工艺陶瓷等“原字号”延伸链条；抢抓大众旅游时代到来、2022年北京冬奥会举办、实施“健康中国”战略等重大机遇，培育战略性新兴产业类项目，推进旅游、冰雪体育运动、节能环保、养老、现代中药等“新字号”发展壮大。对照新一轮东北振兴规划和政策，主动衔接、争取和引进与我市紧密相关的重大项目和资金。优化招商方式，强化招商针对性和务实性。加快绿色食品特色产业园、铜产业园等专业化园区建设。

　　强化农业供给体系建设。以构建现代农业“三大体系”为抓手，以打通供需通道为重点，深入推进农业供给侧结构性改革，提高农产品供给质量，延伸农业产业链，提升价值链，加快发展优质高效农业。在现代农业生产体系上，突出抓好农产品质量。以市场为导向，调好、调顺、调优种植结构。调减玉米，扩大水稻、马铃薯、中草药、杂粮杂豆、果蔬、饲草等优质高效作物种植面积，积极发展食用菌。扩大绿色有机食品基地面积，推进国家级生态原产地产品保护示范区和区域性品牌建设，完善农产品质量监管体系，增加绿色优质农产品供给。提高农业科技应用水平，增强优质农产品生产能力，提升农产品内在价值。新建生态高产标准农田100万亩以上，新增节水灌溉6万亩，治理水土流失10万亩，启动龙江花园水库等项目建设。扩大标准化规模养殖，新建各类标准化规模养殖场65个；推广微生物菌床养殖新技术，创新科学轮牧养殖方式，提高养殖质量、效益和供给力。在现代农业经营体系上，突出抓好农产品流通。引导扶持新型农村经营主体，以市场为导向，由生产型向“种养销”生产经营型转变，增强农产品流通能力。新增生产经营型联合社20个。适应粮食市场化改革，推动粮食仓储企业向“储销”转型发展，完善农产品流通体系。培育壮大农村经纪人队伍，引入国内战略型农产品经营主体，修复农产品流通渠道。加快农村电商发展，优化第三方运营平台战略布局，强化供销社在电商发展中的作用，提高农产品线上交易量。加强地域优质农产品营销，提升价值链。在现代农业产业体系上，突出抓好农产品加工转化。延伸食品产业链条，壮大产业规模，鼓励支持飞鹤、中粮、元盛、恒阳、丰源等现有龙头企业，加快在市域内扩张布局。加快益海嘉里、山东阜丰等战略型项目尽快落地，提高粮食转化率和附加值。大力发展现代畜牧产业，抓好嘉一香生猪屠宰加工、丰源生猪养殖加工、恒阳胶原蛋白复合肠衣、飞鹤奶山羊养殖加工等项目建设。加快马铃薯产业发展，推进希森、中荷等马铃薯加工项目建成投产。推动现代中药产业发展，扩大北药种植和饮片加工基地，引进制药项目，谋划建设北药交易平台。

　　提高工业经济核心竞争力。坚持“抓大不放小”，重点实施“五化”示范工程和中小企业成长工程，千方百计帮助企业降本、提质、增效，提高产品竞争力和有效供给力。实施“五化”示范工程，扩大产品有效供给。围绕高端化，推动产品升级，重点推进一重产品升级改造、齐车重载快捷铁路货车制造等项目。围绕智能化，推动工艺水平提升，重点推进飞鹤智慧工厂群流程制造、华鹤节能门窗数字化升级改造、黑龙冰刀智能车间等项目。围绕绿色化，推动企业绿色低碳发展，重点推进华工机床大力矩节能电机、丰华移动相变蓄能车、百万千瓦级光伏示范基地、“龙瓷·紫陶”产业园循环经济等项目。围绕服务化，推动生产型制造向服务型制造转变，重点推进齐二机床风电机组柔性生产、齐重数控“产品全生命周期管理”、华工机床洁净钢共性技术服务平台和诚成机器人智能焊接生产线等项目。围绕国际化，提高产品国际竞争力，重点推进精铸良与美国西屋公司配套合作、齐车重载快捷货车产品国际资质认证等项目。全年树立市级“五化”示范企业典型10个以上，带动装备制造整机、基础零部件、金属新材料等重点产业发展。深化中小企业成长工程，推动产业集聚。落实好国家和省支持民营经济发展的政策措施，发挥中小企业服务中心作用，在项目审批、融资担保、管理咨询、要素配置等方面创造条件，营造更加优良的市场营商环境。推动中小企业向园区集聚，实施资源整合、分工协作，实现抱团发展。推进中小企业与战略投资者开展股权、技术和营销合作，实现借力发展。引导中小企业主动与域内一重、齐车等龙头企业对接，建立行业联盟，实现配套发展。全市重点培育130户成长型中小企业，力争25户成长为规上企业。促进企业降本增效，激发企业内生动力。积极优化物流配套，争取专项奖补资金和企业直供电政策，降低要素成本；引导企业优化融资结构，加大直接融资，提高贷款周转金和助保金贷款使用效率，降低融资成本；取消和降低一批行政事业性涉企收费，调整失业保险费率和缴费基数，降低制度性交易成本；引导企业完善内部利益分配和绩效责任机制，建立健全现代企业制度，降低企业管理运行成本。

　　加快现代服务业发展。把握产业转型和消费需求变化新趋势，加快发展综合型新业态、智慧型新模式，努力扩大高质量的服务供给，满足需求，引领消费。加快商贸流通新业态发展。坚持上行与下行、引进与培育并重，推进电商产业与实体商贸业融合发展。开展大规模“普及推广式”电商创业培训，新增电商培训基地50个、电商企业90户、网店5000家，上行交易额增长30%。改造提升传统商贸业，限上企业触网率力争超过50%。引进电商企业，整合、服务实体商贸业，限上企业社零额占比提升到30%以上。加快建华恒腾、依安鹏程等商贸物流园区服务升级，支持远赢置业IM交易配送中心等新型商贸物流综合体发展。加快金融产业发展。引入域外金融机构，加快市区信用社改制。完善地方政府融资担保体系，推动社会化担保机构健康发展。创新金融产品，深化银税、银保合作。通过推广、培训、交流等途径，实现“政金企”对接常态化。发挥政府产业引导基金作用，引进风投类基金，加大对企业投贷联动支持力度。推动北方工业股权交易中心“域内百户企业挂牌、场内百户企业股改”,完善北方畜产品交易中心运营功能。严厉打击恶意逃废债，处置好金融债务，防范金融风险。加快旅游业发展。深化与战略投资者合作，提高旅游营销和服务的互联网化水平。突出以鹤为主题的旅游产品市场化开发，推进扎龙生态旅游带、爱飞客航空小镇、奥悦旅游综合体、鹤城欢乐谷、富裕龙腾度假庄园等项目建设。突出冰雪运动、雪地观鹤和寒地温泉等旅游产品融合，加快冰雪旅游产业发展。打造碾子山全域旅游品牌。支持梅里斯民族文化旅游产业发展。打造特色美食街，办好鹤城烧烤美食节和啤酒节。培育旅游专业客运体系，新增一批星级宾馆，建成农家乐基地2处。深化（4+1）城际旅游联盟和东北17城市旅游联盟间交流合作。接待游客和旅游收入分别增长10%和12%。加快特色文化产业发展。整合现有资源，成立市演艺公司，加强与知名文化投资公司合作，进一步活跃文化市场。创作冰上杂技《飞雪迎春》等具有地域文化特色的精品剧目。支持克东满绣、嫩江玛瑙、依安工艺陶瓷等创意文化产业发展。加快冰雪体育运动产业发展。办好冬夏两个冰球季，营造冰雪体育运动产业发展氛围。强化市场化人才培养和赛事服务，形成国内外有影响力的赛事品牌。加快冰上人才培训基地、冰雪装备器材产业园以及冰刀、滑雪板项目建设进度。加快养老产业发展。推进鲜明生态养老中心、嘉祥健康公馆、北方民族老年养生院等项目建设，发展生态型、候鸟式、养医结合式养老产业模式。新增养老床位1000张。扩大智慧社区居家养老范围，深化居家养老社会化服务体系建设。加强养老市场监管，提升养老服务质量。

　　促进区域经济协调发展。统筹总体布局，优化资源配置，增强区域经济发展的协调性和可持续性。促进县域与城区统筹发展。进一步明晰县域和城区经济发展定位，形成“两翼”齐飞、优势互补、错位发展的格局。引导支持县（市）依托资源禀赋，发挥一产特色优势，加快三次产业融合，推动县域经济晋位赶超。坚持市区联动，发挥城区二产带动和三产辐射作用，做大做强主导产业，提升城区经济带动能力。促进央企与地方协同发展。支持央企深化改革、产业升级和人才培养，扩大企业项目和技术溢出。推广一重模式，提高央企本地配套率。促进军民融合深度发展。推动军地经济、科教、人才等各领域深度融合，支持军地产品协同配套。加快碾子山军工旅游等军民融合项目建设。促进地方与高校院所合作发展。建立“校地联盟”科技合作新机制，实现政产学研精准对接。加强与齐大、医学院、省畜研所、哈工大、铁科院等域内外高校院所深度合作，支持科研人员创业，促进产学研成果落地转化。

　　（二）实施“七大民生工程”，着力保障和改善民生。今后五年，坚持以人民为中心，围绕实施“七大民生工程”，持续加大民生投入，解决好群众普遍关心的突出问题，努力让全市人民过上更好的生活。2017年，具体做好以下工作：

　　实施脱贫攻坚工程。把脱贫攻坚作为“一号工程”，做到精准扶贫、精准脱贫。完善脱贫攻坚“1+N”政策体系，通过产业扶贫、金融扶贫、健康扶贫、兜底扶贫等措施，完成131个贫困村、8.1万贫困人口脱贫任务，为2018年末实现贫困人口脱贫和贫困县摘帽奠定基础。

　　实施社会保障工程。稳定就业。落实好就业再就业政策，强化援企稳岗措施，技能培训3.6万人，城镇新增就业8.7万人。提高社保待遇。提高退休职工基本养老金和城乡居民基础养老金标准，出台生活困难群体养老保险助保贷款扶持政策，确保养老金按时足额发放。提高城乡居民医疗保险住院支付限额和报销比例，启动城乡大病补充保险，探索长期护理保险有效实施途径，实施省内异地就医即时结算。强化救助优抚。完善困难群众帮扶救助长效机制，按政策提高城乡低保标准。加快救灾物资储备库等扶贫济困重点项目建设，争创国家级综合减灾示范社区3个、省级4个。抓好公益性养老机构标准化管理，推进荣军养老康复中心和第一福利院智障人员住养楼建设。关爱残疾人，完善孤残儿童保障体系，支持SOS儿童村和市儿童福利院建设发展。新建齐齐哈尔市烈士陵园。做好优抚和“双拥”工作。

　　实施宜居工程。加快棚户区和农村危房改造。加大各县（市）及铁路棚户区改造力度，全市改造棚户区1万户，力争到2020年基本完成棚户区改造。改造农村危房2.9万户。实施老旧小区综合改造。以老旧小区楼体、环境和配套设施为重点，以基本功能修复和提升为目标，改造完成15个老旧小区，为五年左右时间完成全市老旧小区综合改造开好头。提升物业服务质量。开展老旧小区综合整治和物业管理区域整合，强化对物业企业监管，规范物业服务，改善小区环境质量。加强建设工程质量监管，推进房地产市场持续健康发展。

　　实施城市交通改善工程。优化城区路网。编制完成城市综合交通规划。升级改造市政道路91条，其中中心城区27条。完善步行系统和非机动车道路22条。中心城区规划建设停车场12处，提高各类停车设施使用率。加快公交升级。优化公交线路和站点布局，完善运营机制和支持政策，提升公交服务质量。建设公交首末站10处，更新一批公交车辆。提升交通管理水平。优化交通信号系统，增设交通标识，强化交通组织管理，提高道路畅通率。推进公路项目建设。加快南外环、省道江桥至黑蒙界段公路建设，谋划牙四公路碾子山至富拉尔基段公路建设，启动嫩江大桥维修加固工程。北富高速建成通车。修建农村公路300公里。

　　实施城乡环境整治工程。开展“三大防治”行动。严格落实“气十条”“水十条”“土十条”，强化生态红线意识，制定最严格的环境保护措施，实行生态责任审计全覆盖，全力抓好中央和省环保督察组反馈问题的整改落实。淘汰燃煤小锅炉166台、黄标车及老旧车辆9000台，强化燃煤脱硫脱硝措施，提升燃油品质，确保空气质量优良天数达到300天以上；全面建立河长制，抓好饮用水源地保护，推进黑臭水体综合整治，建立乌裕尔河、双阳河等脆弱河流生态保护廊道；加大农村面源污染整治和农业“三减”力度，耕地土壤环境质量达标率不低于90%。开展“四季整治”行动。持续开展好“春风、夏净、秋扫、冬清”整治行动，提高净化、绿化、亮化、美化水平。完善城乡精细化管理责任体系，重点解决背街小巷、居民小区、城乡结合部脏乱差问题。城区栽植树木105万株，乡村植树造林7.5万亩。建设美丽乡村，打造典型示范村189个。提升“三供两治”水平。实施好供热质量提升三年行动，启动富发热源管网配套工程，扩大热电联产集中供热面积，探索新能源供热方式，完成低温楼外墙保温50万平方米。改善供水质量，推进浏园水厂二期工程及新水源建设。加快供热、供水老旧管网改造和随路配套管线建设，优化城市供电线路。启动中心城区防涝排涝专项治理，完成市污水处理厂二期提标改造、污泥处置及餐厨废弃物处置工程，加快固废循环经济静脉产业园建设，推动县级污水处理厂常态化、规范化运行。

　　实施社会事业提升工程。提高教育质量。新改扩建幼儿园20所，建设义务教育标准化学校44所，改造薄弱学校79所，确保3个县区通过国家义务教育基本均衡县验收。深化“双元培育”改革，提升职业教育服务产业能力。开展丰富多彩的校园课外活动，提高学生综合素质。从严整治师德师风。开展“健康鹤城”行动。普及健康知识，倡导健康生活方式。发展医疗联合体，推动医疗服务重心下移，落实基层首诊责任，构建合理就医新秩序，重点人群家庭医生签约服务率达60%。抓好第一医院南院等项目建设。加强公共卫生服务。开展“百万人上冰雪”活动，浇筑冰场170块；新增全民健身器材150套。开展文化惠民活动。恢复龙沙音乐会，办好鹤文化艺术节等群众性文化活动，惠民演出230场。打造中国音乐剧创作基地和中国合唱基地。抓好卜奎清真寺等重点文保工程的管理和修缮工作。

　　实施“平安鹤城”工程。强化安全生产。严格落实安全生产责任制，突出过程监管和隐患整改，坚决遏制重特大事故发生。严格市场监管。建立食品药品质量安全追溯体系，完善学生餐等重点群体食品监管机制，严厉打击制假售假行为。维护社会稳定。创新社会治安综合治理，公共安全和社会管理视频监控实现联网，严厉打击违法犯罪行为。畅通信访渠道，强化信访工作问责，全力解决群众合理诉求。规范管理民间组织。强化社区自我管理、自我服务，推进城乡社区标准化建设，新增城乡示范社区20个。提高社区工作者待遇。深入开展民族团结进步创建活动，依法管理宗教事务。

　　（三）坚持创新驱动，努力打造发展新动能。今后五年，进一步深化改革，扩大开放，深入实施“大众创业、万众创新”，打造发展新动能。2017年，着力在以下方面取得新突破：

　　深化重点领域改革。坚决落实中央和省市委各项改革部署，着力破解体制机制障碍。深化经济领域改革。加快国有企业改革，清查地方国有企业资产，分领域建立资产运营公司，完善市场化国有资本监管体系，分类推进地方国有企业改革，分批推进事转企单位社会保险制度衔接；支持中省属企业深化混合所有制改革，加快“三供一业”移交；解决国有企业历史遗留问题，完成国有厂办大集体改革；全力支持北钢破产重整。深化农业综合配套改革，探索推动农村集体产权制度改革，加快农村土地确权登记颁证，推进土地所有权、承包权、经营权“三权”分置；扩大农村“四权”抵押贷款规模，破解经营主体贷款难题。抓好国有林场改革。开展供销社综合改革试点。深化行政领域改革。坚持“放管服”结合，差异化下沉市级权力；优化审批流程，全面推进“一窗式”政务服务，实现“一号申请、一窗受理、一网办结”；完善商事制度改革，简化后置审批事项，强化事中事后监管；清理国家和省明令禁止的行政审批、职业资格认定和收费事项；完善公共资源统一交易体系建设；深化政府投资项目代建制和集中审查制改革。推进综合执法体制改革，全面实施部门内综合执法，按领域推进联合执法；深化“双随机、一公开”执法体制，推行执法与日常监管分离，控制执法频次，增强执法效果。深化财政体制改革，在完善公共预算、基金预算和国有资本经营预算基础上，全面实施债务预算，强化专项预算，深化公车、接待、出国、用人、资金支付和资产管理等制度改革。深化社会事业和公共服务领域改革。推进事业单位分类改革，加快生产经营类事业单位转企改制。全面实施机关事业单位养老保险制度。深化医药卫生体制改革，完善基本药物制度，加快推进分级诊疗，减轻群众看病就医负担。积极稳妥推进中考改革，适应高考改革。深化文化体制改革，加快与市场和社会融合。

　　扩大对外开放。坚持“出口抓加工、进口抓落地”，强化国际经贸通道建设，推动国际经贸合作，逐步形成对俄欧、东北亚全方位、多层次开放新格局。加强国际贸易平台体系建设。重点推进三间房国际经贸物流产业园区建设，启动基础设施配套工程，加大综合保税区和铁路临时口岸申报力度，争取设立海关检疫集中查验场，加大园区项目引进。以梅里斯中俄优质农产品交互性贸易周转中心为依托，巩固对俄贸易通道，实现农产品往来常态化。加强外向型企业培育。扩大外向型企业规模，加大恒阳、元盛、精铸良、金鼎亚麻、景宏石油等外向型骨干企业扶持力度，鼓励更多企业“走出去”。新增进出口企业20户。加强对外交流合作。深化与国内外友好城市往来交流和经贸合作。落实与广东省内城市建立对口合作机制，实现装备制造优势与东部地区需求有效对接。培育客源市场，开辟新航线，做好机场改扩建前期工作。

　　推动“大众创业、万众创新”。坚持把“双创”作为构建新型产业体系、实现全面振兴发展的最大推动力，让“双创”融入各行业、各领域。加快科技创新。加大科技成果生成和引进力度，提高科技成果转化率。发挥高新区创新孵化龙头作用，建设以“苗圃—孵化器—加速器—园区”为链条的孵化体系，推动平台式科技成果转化。每个县（市）至少新增一个孵化器。新增一批以企业为主体、产学研相结合的技术创新联盟，推动联盟式科技成果转化。加快小微企业技术创新，推动大众式科技成果转化。新生成国家级高新技术企业6户、科技型企业50户。加快推进大众创业。整合各项政策集中向创业发力，引导大学生、城镇职工、科技人员、农民等重点群体，在电商、旅游配套、菌类养殖、文化产业和科研成果转化等领域创新创业。支持大学生在校创业，鼓励科技人员和产业“工匠”领办科技型企业，扶持农民工回乡创业。全市建立大学生创业示范基地10个，创业孵化平台180个。加强民营企业创业者和经营者培训，支持企业家创新创业，引导民营资本领办参建众创空间。今年各县（市）区要新建一批众创空间。加快业态模式创新。以“互联网+”为支撑，推进跨行业融合、产业链融合，打造新型有效供应链体系，发展新经济。以新产业聚集发展为目标，加快特色产业小镇建设，打造规模化创新创业平台。加快人才机制创新。落实好我市《关于深化人才发展体制机制改革的若干意见》，兑现相关待遇和支持政策，加快引进和培养产业实用型人才、经营管理人才、科技人才和专业技术人才，发挥人才在创新创业中的引领作用。

　　（四）加强政府自身建设，切实提高施政能力和服务水平。完成未来五年发展目标及工作任务，必须从严治政。 2017年，主要在以下方面狠下功夫：

　　强化政府效能建设。增强学习研究能力。建立健全政府系统对新经济、新政策、新业态、新模式等专题学习常态化机制，并坚持学以致用，研提推动振兴发展的新建议和新举措。增强改革创新能力。进一步解放思想，鼓励干部创造性地开展工作，以改革创新促发展、惠民生。增强推进落实能力。健全抓落实责任链条和闭环督办制度体系，对重点工作全程跟踪、节点督办、限时问效，坚决整治落实不到位、配合不协调等问题。

　　强化政府法治建设。严格依法行使权力、履行职责，自觉接受人大依法监督、政协民主监督及社会公众监督，办好人大议案、政协提案。启动“七五”普法，认真落实我市法治政府建设实施方案，做好地方性法规草案审修和政府规章制定，提升政府运用法治思维、法治方式办事的能力，时刻绷紧依法行政这根弦。完善政府重大决策、公务运转等运行规则，建立重大决策终身责任追究和责任倒查机制，健全市县乡三级政府法律顾问体系，切实把行政执法责任制落到实处。完善政府信息发布和办事公开制度，扩大公开领域和事项，全面提升政府工作透明度。

　　强化政府诚信建设。把公信力作为政府的生命线，完善政府守信践诺机制，凡是历届市委、市政府依法依规作出的决定和承诺，各级政府及其工作人员必须不折不扣地执行，杜绝“新官不理旧帐”和“上有政策、下有对策”问题，坚决维护政府决策权威和公信力。发挥政府诚信建设的引领作用，带动社会信用体系建设。建立信用信息交换共享机制，全面实行统一社会信用代码和信用信息“双公示”制度，健全守信激励与失信惩戒联合机制，增强全社会以法治、制度、规则为核心的诚信意识。

　　强化政府作风建设。严格落实中央八项规定和省委九项规定，把一切工作的出发点和落脚点都放在勤政为民上，体察民情，了解民意，不负民望，尽职尽责。竭诚为企业服务，积极构建“亲”“清”新型政商关系。严控政府投资项目建设标准、经费使用和地方政府债务，反对“高大上”等形象工程，艰苦奋斗，厉行节约。严控行政编制，缩减事业编制。严控重点领域、重点岗位权力运行。完善岗位责任清单、权力清单和问题清单，建立权力运行监察体系，发挥好监察、审计部门问效、问绩、问责作用。深入落实党风廉政建设主体责任和“一岗双责”，健全廉政建设体系。严守《准则》《条例》等党规党纪，对政府公务人员特别是领导干部行为规范划定红线，从源头上预防腐败。从严惩处以权谋私、弄权勒卡、失职渎职行为，从严整治为官不为、推诿扯皮、办事拖拉问题，持续改善发展环境，努力打造为民、务实、清廉政府。

　　各位代表！新一轮东北老工业基地全面振兴的号角已经吹响，今天的齐齐哈尔正站在新的历史起点上。让我们紧密团结在以习近平同志为核心的党中央周围，在省委、省政府和市委的坚强领导下，万众一心，拼搏进取，奋力推动齐齐哈尔全面振兴发展，以优异成绩迎接党的十九大和省第十二次党代会胜利召开！
